# Stroke and brain atrophy in chronic Chagas disease patients: A new theory proposition

**DOI:** 10.1590/S1980-57642009DN30100005

**Published:** 2009

**Authors:** Jamary Oliveira-Filho

**Affiliations:** MD, PhD, Associate Professor, Coordinator of the Stroke Clinic, Federal University of Bahia. Head of Neurology Service and Neurocritical Care Unit, Hospital Espanhol in Salvador, BA, Brazil.

**Keywords:** Chagas disease, American trypanosomiasis, cognition, cerebrovascular disorders, pathogenesis

## Abstract

Chagas disease (CD) remains a major cause of cardiomyopathy and stroke in developing countries. Brain damage in CD has been attributed exclusively to the effects of structural heart disease on the brain, including cardioembolism and low cardiac output symptoms. However, CD patients also develop stroke and brain atrophy independently of cardiac disease severity. Chronic inflammation directed against T. cruzi may act as a trigger for endothelial damage, platelet activation, acceleration of atherosclerosis and apoptosis, all of which lead to stroke and brain atrophy. In the present article, evidence supporting this new theory is presented, along with considerations towards mechanistically-based targeted treatment.

## Abstract (pt)

A doença de Chagas (DC) permanence uma causa importante de cardiomiopatia e acidente vascular cerebral em países em desenvolvimento. A lesão cerebral na DC tem sido atribuída aos efeitos da doença cardíaca estrutural no cérebro, incluindo cardioembolia e sintomas de baixo débito cardíaco. No entanto, pacientes com DC desenvolvem acidente vascular cerebral e atrofia cerebral independentemente da gravidade da cardiomiopatia. Inflamação crônica direcionada contra o T. cruzi pode agir como desencadeante de lesão endotelial, ativação plaquetária, aceleração de aterosclerose e apoptose, todos predispondo a acidente vascular cerebral e atrofia cerebral. Nesse artigo, as evidências que dão suporte a esta nova teoria são apresentadas, juntamente com argumentos direcionados a um tratamento baseado no mecanismo do dano cerebral.

Chagas disease, also called American trypanosomiasis, is one of the leading causes of cardiopathy and stroke in Latin America.^1-3^ It is caused by a protozoan, *Trypanosoma cruzi*, and transmitted mostly by an insect vector. Between 16 and 18 million individuals are infected worldwide, most in an asymptomatic “indeterminate” form which may last decades before reactivation into a chronic form of tissue inflammation (usually heart, gut and peripheral nervous system), making eradication of the disease extremely challenging.^1^ The numbers are awe-striking: in endemic areas, up to 25% of the population bear positive serologic tests for Chagas disease,^4^ usually infected as children; 30% will go on to develop a chronic form of cardiopathy and 10% will suffer a stroke often as young adults.^5^

In developed countries, most interest in Chagas disease has been directed at two more recent phenomena: the possibility of blood-borne transmission from immigrants of endemic areas;^6-8^ and the resurgence of acute forms of Chagas disease, including encephalitic forms, in patients with HIV co-infection.^9,10^ These forms will not be discussed here. While both are potentially important, the present article will focus on three more general questions relevant to all individuals infected with *T. cruzi*: is there evidence for brain involvement in Chagas disease? If so, what are the mechanisms for this involvement? Finally, what potential treatments exist?

## Historical aspects

At the beginning of the 20^th^ century, Carlos Chagas was a sanitarist tasked with implementing anti-malarial strategies in Minas Gerais state, Brazil. With a curious mind, he heard from the local population of a disease inflicted by an insect bite and proceeded to describe not only the disease, but also its pathogen and mode of transmission. The main characteristics of the disease process were discovered, with an acute phase affecting most organ systems and a high parasitemia; an asymptomatic phase lasting up to 30 years; and a chronic phase with low parasitemia and reactivation of inflammation in two major organ systems, the heart and the gut.^11^

At the time, Carlos Chagas suggested that there might be a chronic encephalitic form of the disease with progressive cognitive abnormalities. This idea was later refuted by other authors,^12,13^ who attributed brain symptomatology and pathological findings not to local inflammation, but to passive cardiopathy-related congestion and brain infarcts.

## Pathological series

Only a handful of pathological series have investigated central nervous system involvement in Chagas disease.^12-18^ In the acute phase, inflammation is intense in the brain, cerebellum and brainstem. Multiple glial nodules occur with inflammation predominating in the white matter. The parasites are found mostly in glial cells, forming intracellular pseudocysts of the amastigote form of the trypanosome. Rare involvement of neurons and brain vessels is also found.

In the chronic cardiomyopathy phase, no parasites were found in the brain in the series described, except for rare patients co-infected by HIV.^9,10^ Most changes are attributable to passive congestion by left ventricular heart failure, low cardiac output, neuronal ischemia, and brain infarcts frequently stemming from a left ventricular apical thrombus. Rare cases of vasculitic and focal encephalitic involvement have also been described, but parasites were found very rarely in these cases of immunocompetent patients.^12,15^

Brain atrophy was also observed by two separate investigators, Alencar and Queiroz.^15-17^ This finding is often overlooked in the literature and deserves further mention. While Alencar attributed brain atrophy to chronic ischemia,^16,17^ Queiroz noted that, when compared to other patients who died with a similar severity of heart involvement due to dilated idiopathic cardiomyopathy, Chagas disease patients more frequently presented with brain atrophy.^13,15^ Although he was unable to find a pathological mechanism for this brain atrophy, Queiroz was the first to note that further factors other than structural heart disease must be involved.

Other case series identified a unique population: patients with a history of Chagas disease who died of ischemic stroke, but without clinical or pathological signs of cardiac disease.^19^ Since these patients died without a thorough investigation of stroke etiology, the cause of ischemic stroke in these cases remains unknown. Another limitation of previous pathological series was that no specific immune staining technique looking for more subtle, chronic inflammation was used.

## Stroke and Chagas disease

Dilated cardiomyopathy due to Chagas disease is a highly embolic disease. Intracardiac thrombus is found in 36%20 to 76%^21^ of autopsies, and in 23% of patients in a recent transesophageal and transthoracic echocardiographic study.^22^ Stroke is a major cause of death and incapacity in these patients, occurring in 10% to 20% of patients.^5,22^ These figures may be an underestimation, since microembolism may lead to mild cognitive dysfunction and not a more obvious acute stroke syndrome. In a recent study, our group detected microembolic signals on transcranial Doppler monitoring more frequently in Chagas disease patients than in patients with other cardiomyopathies (16.3% vs. 2.2%, *p<*0.05).^23^

Despite the obvious association between cardiac disease and ischemic stroke in Chagas disease, we and others have found patients with non-cardioembolic stroke mechanisms.^5,19,24,25^ In these patients, chronic inflammation due to Chagas disease may be sufficient to trigger endothelial dysfunction, platelet activation, the coagulation cascade, atherogenesis or a combination of the aforementioned mechanisms. Recently, using microarray analysis, we have detected activation of various genes in peripheral blood leukocytes associated with inflammation, atherogenesis and apoptosis, which are more frequently seen in Chagas disease patients compared to controls.^26^

Thus, four additive mechanisms may contribute to ischemic stroke risk in Chagas disease:

1) cardioembolism causing classical acute stroke syndromes;
2) microembolism potentially causing cognitive dysfunction;
3) chronic inflammation leading to atherogenesis or activation of the coagulation system; and
4) less frequently, watershed infarcts from low cardiac output states.

## Brain atrophy

As previously mentioned, pathological series raised the question of whether brain atrophy could be directly related to Chagas disease.^15-17^ However, this phenomenon may have been mere chance, as these series could not correct for known confounders of brain volume such as age, gender, co-morbidities, alcohol use, duration and severity of cardiomyopathy. Thus, we have recently compared volumes of brain, cerebellum and ventricles in 41 patients with Chagas disease and 32 controls with other cardiomyopathies matched for age and gender.^27^ These patients also had similar cardiac disease severity and duration. Brain volume was a mean 15% lower in Chagas disease patients (*p*<0.001). However, these patients harbored the same number of brain infarcts.

What, then, is the mechanism for this brain atrophy? Recently, normal volunteers in the Framingham Heart Study were submitted to magnetic resonance imaging scans.^28^ In these individuals, high levels of TNF-a and IL-6, both markers of systemic inflammation, were found to be associated with brain atrophy. These same markers have been shown to be increased in patients with chronic chagasic cardiomyopathy^29-31^ and may represent a link between chronic inflammation and a progressive form of brain atrophy.

## Cognitive dysfunction

Despite the previous data showing brain involvement due to infarcts and atrophy, few groups have formally investigated cognitive function in Chagas disease. We found only one previous study on cognitive function in Chagas disease, in which the authors compared Chagas patients with normal controls.^32^ Several cognitive domains were affected, such as non-verbal reasoning, orientation, problem-solving and sequencing. However, these same abnormalities may be due to the presence of cardiomyopathy and its effects on the brain (i.e., from hypoperfusion or brain infarcts).

Another study evaluated 27 patients with either indeterminate or stage A cardiac form of Chagas disease (without structural cardiac damage) and an equal number of controls.^33^ Unspecific white matter demyelination and electroencephalographic abnormalities were found but not associated with significant cognitive dysfunction. Brain atrophy was not described in this study.^33^

In the present volume of **Dementia & Neuropsychologia**, we have published the first formal cognitive evaluation of Chagas disease patients controlling for the presence of cardiac disease, with the disease being the same in cases and controls alike.^34^ Subtle abnormalities of visuo-spatial and visual memory deficits were detected. We have recently expanded the cognitive battery to include the clock drawing test and Luria’s sequence and have found both to be more frequently abnormal in Chagas disease patients clinically unaffected by stroke (unpublished observation).

## New theory proposition

Why propose a new theory? The short answer is that the current theory does not explain the type of brain involvement we and others see in Chagas disease patients. Current theory suggests that all brain involvement in Chagas disease is due to heart-related phenomena.

**Figure 1:**
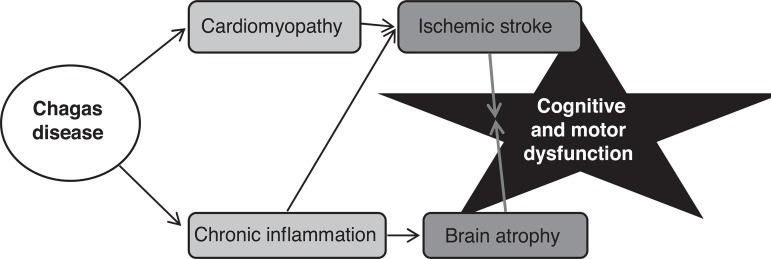
Proposed theory for brain dysfunction in Chagas disease. Chagas disease causes both structural heart damage and chronic activation of the immune system, mostly by Th1-type cytokines. Structural cardiomyopathy and chronic inflammation exert independent and synergistic effects on ischemic stroke risk, while chronic inflammation may induce brain atrophy. Finally, both multiple brain infarcts and brain atrophy impact brain dysfunction such as motor and cognitive deficits.

We propose that brain involvement in Chagas disease may be due to two main non-competing mechanisms (Figure 1). The first and probably most frequent involves structural heart damage from *T. cruzi*, formation of intracardiac thrombosis and embolization to the brain. The second co-existing mechanism involves the immune response to *T. cruzi*. Chronic inflammation, acting mostly by Th1-type cytokines (high TNF-γ and IFN-γ, low IL-10),^29-31,35^ may accelerate atherosclerosis leading to ischemic stroke, and induce apoptosis leading to brain atrophy. Both structural heart disease and chronic inflammation impact cognition and stroke risk, but have potentially very different treatments.

## Treatment considerations

Current treatment paradigms directly reflect the classic view of passive brain involvement due to heart disease. Ischemic stroke due to Chagas disease is typically treated with oral anticoagulation (warfarin). Both “silent” infarcts and cognitive dysfunction go unnoticed and untreated.

We agree that patients with Chagas disease cardiomyopathy who suffer an ischemic stroke should be anticoagulated with warfarin, as should any other patient with other etiologies of dilated cardiomyopathy (e.g., ischemic, idiopathic). However, it is among the patient group without evidence of structural heart damage or embolic arrhythmias; or patients with cognitive dysfunction associated with brain atrophy, that no effective treatment has been tested at all. Should these patients be receiving anticoagulant, antiplatelet, anti-inflammatory, immune modulation or combined treatments? Current uncertainties are the perfect setting for high quality clinical trials. We owe this to our patients.
